# Investigation of novel autoantibodies in Sjogren’s syndrome utilizing Sera from the Sjogren’s international collaborative clinical alliance cohort

**DOI:** 10.1186/s12886-015-0023-1

**Published:** 2015-04-10

**Authors:** Lakshmanan Suresh, Kishore Malyavantham, Long Shen, Julian L Ambrus

**Affiliations:** Immco Diagnostics, Buffalo, NY USA; Department of Medicine, SUNY at Buffalo School of Medicine, Buffalo, NY USA; Division of Allergy, Immunology and Rheumatology, SUNY at Buffalo School of Medicine, Room C281, Buffalo General Hospital, 100 High Street, Buffalo, NY 14203 USA; Department of Oral Diagnostic Sciences, SUNY at Buffalo School of Dental Medicine, 3435 Main Street, Buffalo, NY 14214 USA

**Keywords:** Sjogren’s syndrome, Dry eyes, Autoantibodies

## Abstract

**Background:**

Sjogren’s syndrome (SS) is a chronic autoimmune disease mainly affecting salivary and lacrimal glands. Current diagnostic criteria for SS utilize anti-Ro and anti-La as serological markers. Animal models for SS have identified novel autoantibodies, anti-salivary gland protein 1 (SP1), anti-carbonic anhydrase 6 (CA6) and parotid secretory protein (PSP). These novel antibodies are seen in the animals at an earlier stage of SS than anti-Ro and anti-La. The current studies were designed to evaluate these novel autoantibodies in the sera of well-characterized patients with dry eyes and dry mouth and lip biopsies from the Sjogren’s International Collaborative Clinical Alliance (SICCA) to determine if they indeed identify SS with less severe disease than patients expressing anti-Ro and anti-La.

**Methods:**

Sera were obtained from SICCA registry in patients for whom lymphocytic foci per 4 mm^2^ on the lip biopsies was either 0 (F = 0), <1 (F <1) or > 3 (F >3). ELISA assays were utilized to evaluate these sera for anti-Ro, anti-La, anti-SP1, anti-CA6, and anti-PSP.

**Results:**

In patients with dry eyes and dry mouth but F = 0, increased expression of anti- CA6 was noted compared to the F <1 group (p = .032) or the F > 3 group (p = .006). Neither anti-PSP nor anti-SP1 reached statistical significance because of the small numbers in the F0 group, although there was a trend for their expression to be higher in the F0 group. On the other hand, the expression of anti-Ro was significantly reduced in the F0 group compared to the F <1 (p = .0021) and F > 3 (p = .0003) groups. The reduced expression of anti-La in the F0 group compared to the F <1 and F > 3 groups did not quite reach statistical significance.

**Conclusions:**

Anti-Ro and anti-La identify patients with SS and more severe disease than anti-SP1, anti-CA6, and anti-PSP. More studies are needed to identify the timing in the course of SS when these different autoantibodies are expressed and/or whether they are expressed in patients with different clinical manifestations.

## Background

Sjogren’s syndrome (SS) is a common autoimmune disease characterized by dry eyes and dry mouth along with associated clinical manifestations that can include interstitial lung disease, kidney disease, neuropathy, vasculitis and lymphoma [1,2]. Therapeutic trials in SS have emphasized the importance of early diagnosis for optimal response to therapy [3]. Current diagnostic criteria both from the American College of Rheumatology and the American-European Consensus Group utilize anti-Ro and anti-La [4]. Several studies have emphasized the existence of many patients with SS lacking these markers. Recently studies with animal models of SS have identified novel autoantibodies, anti-salivary gland protein 1 (SP1), anti-carbonic anhydrase 6 (CA6) and anti-parotid secretory protein (PSP) [5]. These autoantibodies were shown to be present in patients with SS as well as in patients with idiopathic dry mouth and dry eyes [5,6]. The current studies were undertaken to investigate expression of these autoantibodies in the sera of well-characterized patients with different levels of focus score (FS) measured from labial salivary glands and used as a possible indicator of SS disease severity.

## Methods

### Sera

Sera were obtained along with demographics of the patients from the Sjogren’s International Collaborative Clinical Alliance (SICCA). SICCA is an ongoing longitudinal multi-site observational study that is studying a large cohort of uniformly evaluated individuals from ethically diverse populations. SICCA participants must be at lest 21 years of age and have: a complaint of dry eyes or dry mouth or a previous suspicion or diagnosis of SS or elevated serum ANA, RF, SS-A, or SS-B, or bilateral parotid enlargement in a clinical setting of SS, or a recent increase in dental caries [7]. Patients signed informed consent when participating in SICCA but did not sign a specific informed consent for these studies. Patients sera for this study were requested based on FS, FS = 0, FS < 1 per 4 mm^2^ and FS > 3 per 4 mm^2^ to select patients with different degrees of salivary gland inflammation.

Normal controls were obtained from donors in Buffalo, New York lacking defined medical illnesses, dry eyes or dry mouth. Of the patient groups studied the FS = 0 contained 9 such patients of whom 4 were males and had a mean age of 49.8 years. In the FS < 1 group there were 40 patients of whom 3 were male and there was a mean age of 49.9 years. The FS > 3 group had 40 patients of whom 2 were males and had a mean age of 51 years. There were 50 normal controls that included 5 males with a mean age of 37.5 years. Approval for these studies was obtained from the Institutional Review Board, SUNY at Buffalo School of Medicine.

### Assays

Autoantibodies to SP1, PSP, CA-VI, Ro and La were determined using ImmuLisa ELISAs (Immco Diagnostics Inc., Buffalo, NY). In brief, kits contain micro-wells coated with highly purified recombinant antigen (SP1 or PSP or CA-VI or Ro or La) or native antigens (RF). 96 plate wells are blocked and stabilized by the manufacturer to reduce non-specific binding. Controls, calibrators and diluted (1:100) patient serum are incubated in the antigen-coated wells to allow specific binding of autoantibodies to the antigen. Unbound serum components are washed off and bound antibodies are detected after incubations with anti-human IgG conjugated to HRP (Horse Radish Peroxidase) and specific enzyme substrate (TMB) as per manufacturer’s protocol. The chromogenic reaction is stopped and the intensity of the color change, which is proportional to the concentration of the bound autoantibody, is read by a spectrophotometer at 450 nm. Results are expressed in ELISA units per milliliter (EU/ml) using the calibrators provided in the kit and reported as positive for a value > 20 or negative for a value <20.

### Statistics

Data were analyzed using unpaired student’s t tests with Prism 6 software (GraphPad, La Jolla, CA).

## Results and discussion

**Figure 1:**
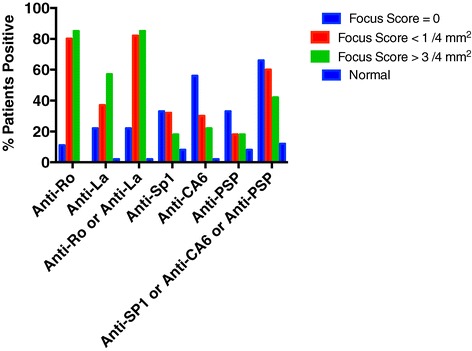
**Sera were obtained from SICCA on patients with complaints of dry eyes and dry mouth who had lip biopsies with focus scores = 0 (9 patients; 4 male, mean age 49.8 years), focus scores < 1 / 4 mm** ^**2**^ **(40 patients; 3 male; mean age 49.9 years) or focus scores > 3 / 4 mm** ^**2**^ **(40 patients; 2 males; mean age 51 years).** Normal controls contained 5 males and had a mean age of 37.5 years (50 patients). In the anti-CA6 studies, positives ranged from 20.7 – 46.4 and negatives from 0 – 19.6. In the anti-PSP studies, positives ranged from 20.3 – 99 and negatives from .1 – 14.6. In the anti-SP1 studies, positives ranged from 20.7 – 65.1 and negatives from 0 – 17.1. In the anti-Ro studies, positives ranged from 26.2 – 330 and negatives from 1.1 – 16.6. In the anti-La studies, positives ranged from 20.9 – 195. 4 and negatives from 0.9 – 19.6. In each of the study groups, F = 0, F <1 and F > 3, the expression of the autoantibodies, anti-CA6, anti-PSP, anti-SP1, anti-Ro and anti-La, was statistically significant when compared to the normal controls (in all cases p < .0005). The increased expression of anti- CA6 in the F0 group compared to the F <1 group (p = .032) or the F > 3 group (p = .006) were statistically significant. Neither anti-PSP nor anti-SP1 reached statistical significance because of the small numbers in the F0 group, although there was a trend for their expression to be higher in the F0 group. On the other hand, the expression of anti-Ro was significantly reduced in the F0 group compared to the F <1 (p = .0021) and F > 3 (p = .0003) groups. The reduced expression of anti-La in the F0 group compared to the F <1 and F > 3 groups did not quite reach statistical significance.

Studies utilizing the IL-14α transgenic (IL14aTG) mouse model of SS demonstrated that loss of salivary gland function occurs before infiltration of the submandibular and lacrimal glands with lymphocytes [8,9]. Furthermore, anti-SP1 and anti-CA6 were demonstrated in the sera of the mice at 6 months of age, a time in which salivary gland function was lost but no significant lymphocytic infiltration was noted in the glands [5]. We were therefore interested in studying patients with dry eyes and dry mouth lacking lymphocytic infiltration of the salivary glands. The SICCA cohort contained 9 such patients with lip biopsies described as showing sclerosing chronic sialoadenitis but no evidence of lymphocytic infiltration. The group contained 4 males and had a mean age of 49.8 years. At the same time, we studied 40 sera from patients with mild disease (focus score < 1 per 4 mm^2^) and 40 patients with moderate disease (focus score > 3 per 4 mm^2^). The group with mild disease contained 3 males and had a mean age of 49.9 years. The group with moderate disease contained 2 males and had a mean age of 51 years. There were 50 normal controls that included 5 males with a mean age of 37.5 years. Figure 1 demonstrates that in the patients with focus score = 0, more patients expressed antibodies anti-SP1, anti-CA6 and anti-PSP than anti-Ro and anti-La (6 vs 2). In patients with mild (F <1) or moderate disease (F >3), the majority of the patients expressed anti-Ro or anti-La while fewer numbers of patient sera contained anti-SP1, anti-CA6 and anti-PSP. In each of the study groups, F = 0, F <1 and F > 3, the expression of the autoantibodies, anti-CA6, anti-PSP, anti-SP1, anti-Ro and anti-La, was statistically significant when compared to the normal controls (in all cases p < .0005). The increased expression of anti- CA6 in the F0 group compared to the F <1 group (p = .032) or the F > 3 group (p = .006) were statistically significant. Neither anti-PSP nor anti-SP1 reached statistical significance because of the small numbers in the F0 group, although there was a trend for their expression to be higher in the F0 group. On the other hand, the expression of anti-Ro was significantly reduced in the F0 group compared to the F <1 (p = .0021) and F > 3 (p = .0003) groups. The reduced expression of anti-La in the F0 group compared to the F <1 and F > 3 groups did not quite reach statistical significance.

**Figure 2:**
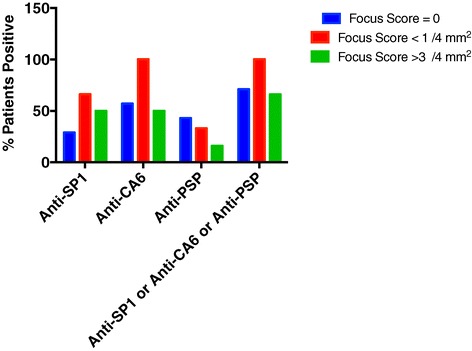
**Patients from the SICCA Cohort lacking antibodies anti-Ro or anti-La were evaluated for their expression of anti-SP1, anti-CA6 and anti-PSP.** In the focus score = 0 group there were 7 patients, in the focus score < 1 / 4 mm^2^ there were 3 patients and in the focus score > 3 / 4 mm^2^ there were 6 patients. Data shown are the percentage of patients positive for the designated autoantibodies. The patient numbers are too small for statistical significance to occur in the differences between the expression of the various autoantibodies.

In the focus score = 0 group, there were 7 patients lacking anti-Ro or anti-La. In the F <1 group 3 patients lacked anti-Ro or anti-La while in the F > 3 group 6 patients lacked these autoantibodies. Figure 2 demonstrates that in the anti-Ro or anti-La negative patients, most patients expressed anti-SP1, anti-CA6 or anti-PSP, although there were a few patients who expressed none of these autoantibodies in the focus score = 0 and the moderate groups. Because of the small numbers, these observations do not reach statistical significance. More patients need to be studied to confirm these trends.

Our understanding of the pathophysiology of SS is currently in flux. While early models of SS had suggested a disease largely driven by the adaptive immune system, more recent models have implicated the role of the innate immune system in the initiation of the disease [10]. Of note, in the IL14aTG model for SS the innate immune system is responsible for the majority of the destruction of the salivary and lacrimal glands [11,12]. Lymphocytes are found in the salivary and lacrimal glands only after significant loss of salivary gland function [12]. In this model, antibodies anti-SP1, anti-CA6 and anti-PSP occur during this early stage of disease, while antibodies anti-Ro and anti-La occur later in the disease course [5].

The data presented in this manuscript suggest that patients with low FS on salivary gland biopsies express anti-SP1, anti-CA6 and anti-PSP more frequently than anti-Ro and anti-La, just like the IL14aTG mice. The FS = 0 group, however, does not meet full criteria for SS, so they would not officially be given that designation. There are no data available to determine whether this group ever will meet full SS criteria. Previously published studies have shown frequent expression of anti-SP1 in patients with idiopathic dry eyes and dry mouth for less than 2 years [5]. What is unclear from this work is how many of these patients in time will meet full criteria for SS and how many of these patients will not progress further.

Interestingly, a recent study from Jonsson et al. demonstrated the existence of anti-Ro or anti-La in the sera of patients often before they developed clinical evidence of SS [13]. Similar finding were made in a previous study in SLE [14]. Many patients who developed SS, however, did not express anti-Ro or anti-La. Antibodies anti-SP1, anti-CA6 and anti-PSP were not evaluated in these studies.

One of the novel aspects of the antigens SP1, CA6 and PSP compared to Ro and La is that they are found selectively in the salivary and lacrimal glands. Ro and La are found virtually in every cell. It is unclear why antibodies anti-Ro and anti-La should be specific for SS, and in fact they are found in SLE and various other autoimmune diseases. Perhaps certain types of cellular injury are necessary before antibodies anti-Ro and anti-La appear. It has been postulated that SP1, CA6 and PSP may have roles in the adherence and/or clearance of various infections [15]. If this were in fact the case, it would be logical to suspect that a hapten-carrier system established with an infectious agent could lead to the development of antibodies to these antigens. If we postulate that SS is initiated by infections in the salivary and lacrimal glands, production of antibodies anti-SP1, anti-CA6 and anti-PSP early in the course of disease would make sense.

The role of autoantibodies in SS is currently poorly understood. While vaccination of mice with Ro results in salivary gland injury and anti-Ro antibodies, it is unclear whether the anti-Ro antibodies participate in the salivary gland injury, are part of a reparative mechanism for the injury and/or are merely a marker for disease [16]. Attempts have been made to correlate autoantibodies with particular disease manifestations in SS, such as anti-carbonic anhydrase 2 antibodies with renal tubular acidosis, however no direct link has been made between the autoantibodies and tissue dysfunction [17]. Similarly, the anti-SP1, anti-CA6 and anti-PSP antibodies are currently evaluated only as markers of disease. Future studies will need to investigate their significance.

While these studies suggest that anti-SP1, anti-CA6 and anti-PSP are early markers for SS, future studies will have to evaluate these autoantibodies in other cohorts of patients. It will be necessary to determine the long-term consequences of expression of these autoantibodies in particular patients and “normal controls”. These autoantibodies may denote particular stages of SS and/or particular forms of SS.

## Conclusions

Novel autoantibodies, anti-SP1, anti-CA6 and anti-PSP, identify patients with dry eyes and dry mouth and low focus scores on lip biopsies while anti-Ro and anti-La, which are currently in the diagnostic criteria for Sjogren’s syndrome occur more in patients with high focus scores on lip biopsy than in patients with low focus scores on lip biopsy.
